# A continuous-time Markov chain model of fibrosis progression in NAFLD and NASH

**DOI:** 10.3389/fmed.2023.1130890

**Published:** 2023-05-30

**Authors:** Lyndsey F. Meyer, Cynthia J. Musante, Richard Allen

**Affiliations:** Pfizer Worldwide Research Development and Medical, Cambridge, MA, United States

**Keywords:** NAFLD, NASH, fibrosis progression, Markov chain model, liver biopsy, fibrosis score

## Abstract

The specific pathways, timescales, and dynamics driving the progression of fibrosis in NAFLD and NASH are not yet fully understood. Hence, a mechanistic model of the pathogenesis and treatment of fibrosis in NASH will necessarily have significant uncertainties. The rate of fibrosis progression and the heterogeneity of pathogenesis across patients are not thoroughly quantified. To address this problem, we have developed a continuous-time Markov chain model that is able to capture the heterogeneity of fibrosis progression observed in the clinic. We estimated the average time of disease progression through various stages of fibrosis using seven published clinical studies involving paired liver biopsies. Sensitivity analysis revealed therapeutic intervention at stage F1 or stage F2 results in greatest potential improvement in the average fibrosis scores for a typical patient cohort distribution. These results were in good agreement with a retrospective analysis of placebo-controlled pioglitazone clinical trials for the treatment of NAFLD and NASH. This model provides support for determining patient populations, duration, and potential successful endpoints for clinical trial design in the area of NAFLD and NASH.

## 1. Introduction

Nonalcoholic Fatty Liver Disease (NAFLD) is the most common liver disease in the United States. In approximately 20% of the affected population, NAFLD progresses to nonalcoholic steatohepatitis (NASH) the hallmarks of which are inflammation, hepatocellular ballooning, and subsequent worsening fibrosis (1). Left untreated, NASH can ultimately progress to cirrhosis of the liver and hepatocellular carcinoma. NAFLD and NASH are the root cause of ~30% of all liver transplants in the United States (2). Currently, there are no FDA approved treatments for NAFLD or NASH resulting in a substantial unmet medical need.

It is hypothesized that liver injury is initiated following excess hepatic lipid accumulation leading to oxidative stress and inflammation (3). However, the complex dynamical relationships between the key biological pathways and processes leading to fibrosis are not well understood. Hepatocyte stress, ballooning, and death contribute to the recruitment of macrophages (4). Macrophages potentiate collagen deposition *via* hepatic stellate cell activation through cytokines, such as transforming growth factor-beta (TGFβ) and platelet-derived growth factor (PDGF) (5). A particular challenge in understanding the pathogenesis is that some patients exhibit rapid disease progression while others progress more slowly, and some portion of the observed population may exhibit stable disease or improve (6).

Currently, liver biopsy is considered the “gold standard” for the clinical diagnosis of NAFLD and NASH (7). Trained pathologists analyze biopsy samples and score fibrosis progression according to the criteria specified by Brunt et al. in Table 1 (8, 9). The invasive nature of biopsy and the histological assessment of fibrosis each contribute to significant challenges in evaluating the time course of disease progression. Due to the risks involved with obtaining a liver biopsy, it is often difficult to obtain multiple biopsies from a single patient and in cases where a patient’s fibrosis score is in stage 4 it may be unethical to resample. Additionally, there are several sources of variability associated with histological assessment, including variability from the area of the liver sampled, as well as the expertise of the pathologist and the discrete nature of the fibrosis score (7). In addition, the scoring can be subjective, for example, even an experienced pathologist may score the same histological sample differently on the same day.

**Table 1:** Fibrosis scoring definitions.

Stage	Fibrosis
F0	None
F1	Zone 3 Perisinusoidal
F2	F1 + Periportal
F3	Bridging
F4	Cirrhosis

Fibrosis scoring definitions summarized from Kleiner and Brunt et al. (8).

The objective of this work is to develop a computational model capable of capturing the time-course of fibrosis progression and the associated variability in order to provide insights into successful clinical trial design. Given the discrete scoring system and variability of the observed data, we chose to develop a continuous-time Markov chain (CTMC) model. Examples from the literature highlight the application of CTMC models to describe disease progression, such as renal function and hepatitis C (10, 11). In a CTMC model, there are defined states and transition rates, which determine the time and the next state. In our case, the discrete states correspond to fibrosis stage. Markov chain models are probabilistic and time independent. The critical property is that the model is memoryless; that is, the probability of changing states in a given interval is fixed. Therefore, the model requires no assumptions about the previous state of the “agent” (in this case, a patient) and the probability of disease progression or improvement is the same within a given time interval. Here, we describe model development and an application of the model to quantify pioglitazone effects on fibrosis progression as a proof of principle, as well as a power analysis to aid in clinical trial design. Pioglitazone is hypothesized to improve lipid metabolism and insulin sensitivity *via* PPARγ agonism and has been studied in clinical trials as a potential treatment for NAFLD (12).

## 2. Methods

### 2.1. Model development

**Figure 1:**
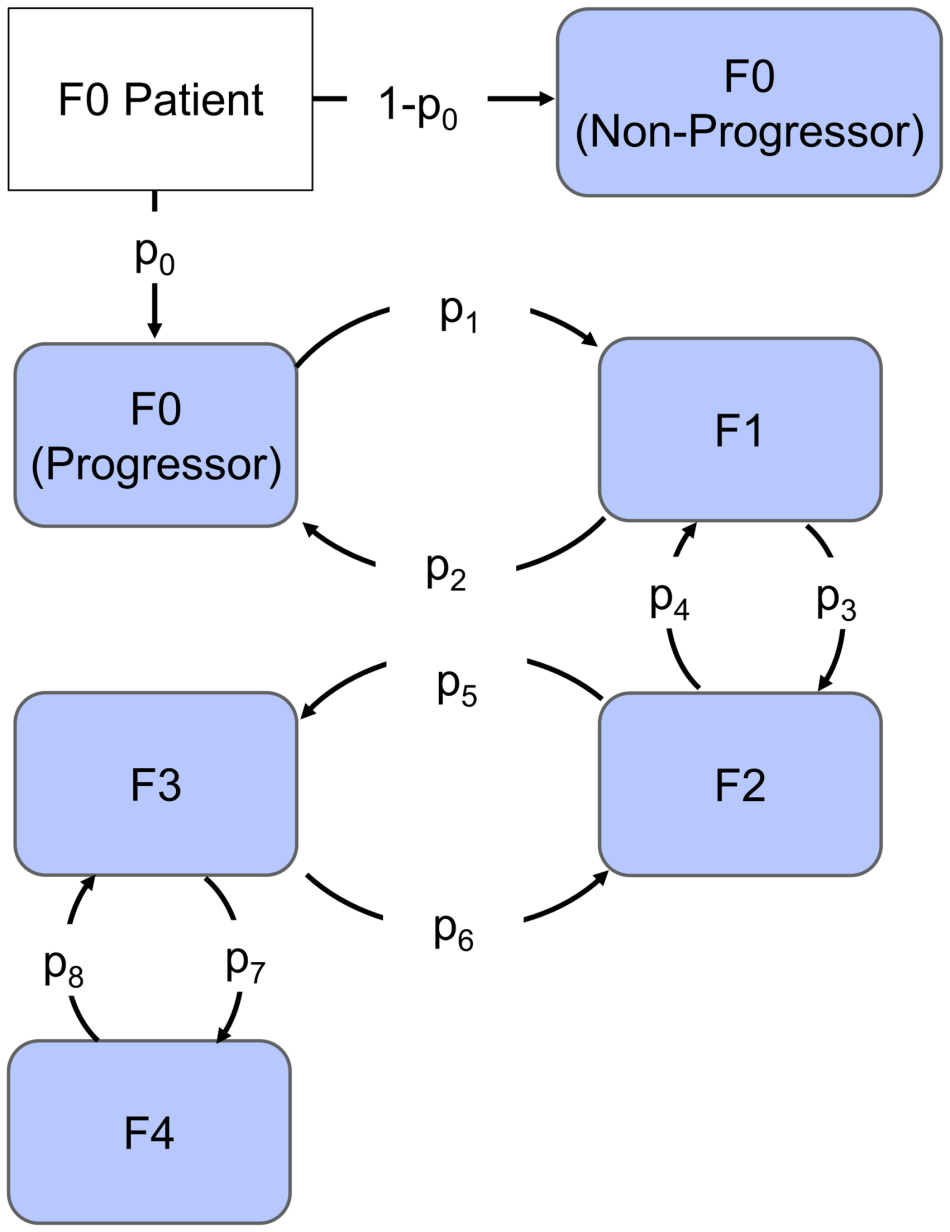
Continuous-time Markov chain model. Each compartment represents the stage of Fibrosis from F0 to F4. The arrows represent rates of progression (p1, p3, p5, and p7) or regression (p2, p4, p6, and p8), the fraction that progresses is indicated by parameter p0 and non-progressors are represented by the remaining fraction.

Given the challenges associated with quantifying the specific mechanisms governing fibrosis progression as well the discrete nature of fibrosis clinical assessment, we chose to employ a continuous-time Markov chain model shown in Figure 1. There are five potential states of the model representing each stage of fibrosis. Each subject scored at F0 was designated as a progressor or non-progressor depending on the probability of progression parameter (*p*_0_) estimated by the model. Progressors move through the various stages of fibrosis with a probability of progression or regression that is independent of how long the subject was in that stage of fibrosis. These characteristics allow us to capture the heterogeneity of clinical cohorts, as well as variability due to different biopsy sampling regions, and histological scoring variations between studies.

To simulate the model, we used a next-reaction, τ leaping algorithm from Thanh et al. in which the reaction rate, τ , is assumed to be a constant. The firing time is then drawn from an exponential distribution where r is a random number from 0 to 1 (13).


(1) $$\tau =\frac{1}{\lambda }\ln\left(\frac{1}{r}\right)$$

At each time point, the algorithm determines the τ for both the forward and reverse reactions and chooses the state associated with the shortest reaction time. Parameters (p_1_–p_8_) governing transition between states (Figure 1) represent the average reaction rate; hence, the reciprocal of a parameter is the average time to disease progression or improvement event. The timescale of the model was chosen to be months based on the frequency of sampling and duration of clinical studies. Model development and parameter estimation was carried out in MATLAB (v. R2019b, Mathworks, Natick, MA, United States). We employed a genetic algorithm with a maximum population of 200 and maximum number of generations up to 100 to identify parameter estimates. In order to compare the final model to the data, we simulated the initial distribution 100 times and averaged the results. Parameters were fit with a sum of squared error objective function weighted by the number of patients at each time point. Simulation time is on the order of minutes for a single trial simulation. Complete MATLAB (v.2019b, Mathworks, Natick, MA, United States) model code is available at https://github.com/pfizer-opensource/CTMC-NAFLD-fibrosis.

### 2.2. Disease progression data fittings

A literature search identified published clinical studies in which patients had biopsy proven NAFLD or NASH. The studies chosen for further analysis were those reporting both the initial number of patients in each stage of fibrosis as well as the final distribution for each initial stage. Studies were included for model fitting if patients underwent paired liver biopsy; the time between biopsies was clearly defined; and the fibrosis scoring was done according to Kleiner or Brunt scoring. As a result, data for fitting was acquired for 6, 24, 36, 48, 60, 72, 96, and 156 months for a total of 218 patients. Data were sourced from Harrison et al., Ratzui et al., Wong et al., Hui et al., Chan et al., Evans et al., and Ekstedt et al. (14–20). Patient demographics varied slightly between studies but most included patients with an average age of 47 years, BMI greater than 25 kg/m^2^, and a mixture of patients with and without diabetes (14–20). All studies excluded patients with excessive alcohol consumption in the last 2 years as well as patients testing positive for hepatitis B surface antigen or anti-hepatitis C virus antibody; patients with secondary causes of hepatic steatosis were also excluded. Data were fit simultaneously using the methods described above.

### 2.3. Sensitivity analysis

Next, we performed a sensitivity analysis to identify which parameters have the most influence on the average change in fibrosis score. In this scenario, we chose to use an initial distribution from a clinical trial of pioglitazone (21). We then simulated the outcome of the trial 500 times and quantified the average change in fibrosis score resulting from a change to a single parameter from 0.001 to 100-fold. We conducted the simulation for each parameter and plotted the change in average fibrosis score +/− the standard deviation vs. the parameter fold-change corrected by the placebo change in fibrosis score. The most sensitive parameters altered the change in average fibrosis score the most.

### 2.4. Pioglitazone intervention

Following the sensitivity analysis, we assessed how an intervention such as pioglitazone impacts both the forward and reverse model parameters and compared the results with the sensitivity analysis. We again performed a literature search to identify published clinical data that included a placebo and pioglitazone treatment group with paired liver biopsies. Three studies met this inclusion criteria, including Cusi et al., Belfort et al., and Aithal et al. (21–23). The doses ranged in each study from 30 to 45 mg/day of pioglitazone. The clinical trial endpoints were assessed at 6, 12, and 18 months for Belfort et al., Aithal et al., and Cusi et al. data, respectively. Data reported by each of these studies did not track individual patient starting and end fibrosis stages; only the initial distribution and final distribution were reported for each group as shown in Table 2.

**Table 2:** Pioglitazone fibrosis data extracted from Appendix Table 1 in Cusi et al. (13).

Stage	Placebo	18 months	Pioglitazone	18 months
F0	20	18	15	22
F1	22	16	22	13
F2	4	3	6	2
F3	5	5	7	3

**Figure 2:**
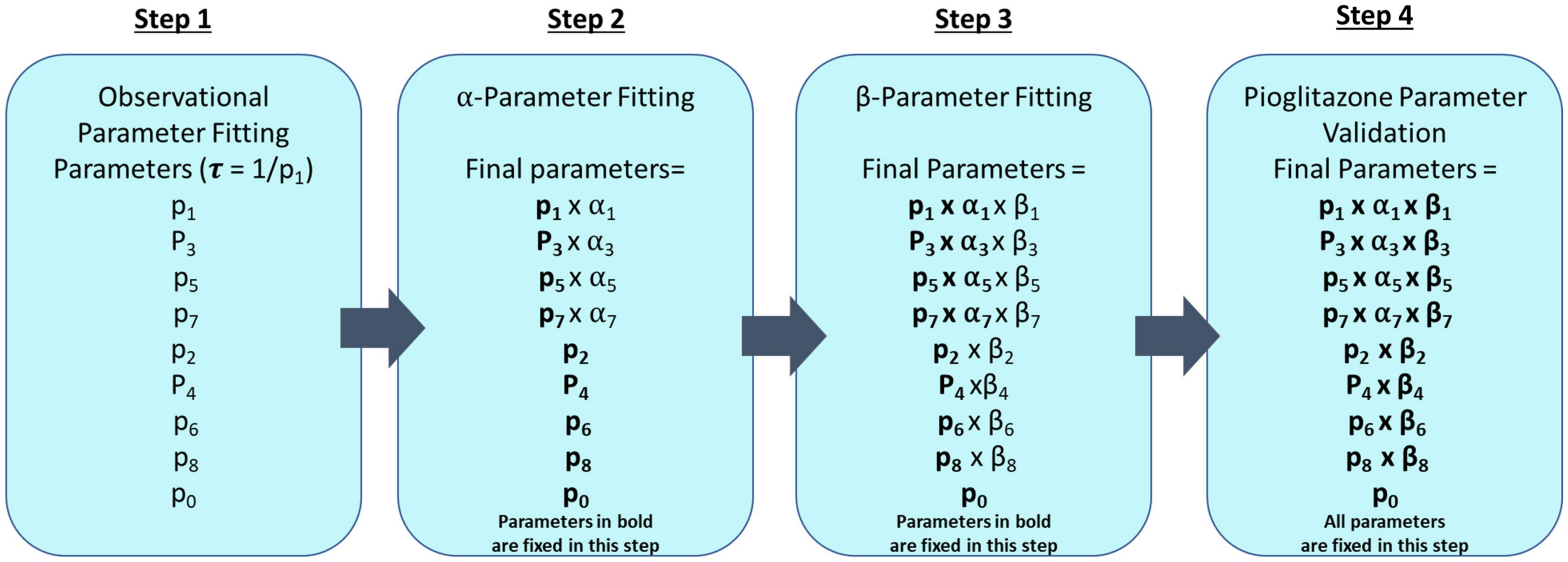
Parameter fitting flowchart. This diagram describes each step that was taken to fit CTMC model parameters where fixed parameters are in bold type. The first step fits all forward (p1, p3, p5, and p7) and reverse parameters (p2, p4, p6, and p8) and progression fraction (p0) to observational data from paired liver biopsy studies Harrison et al., Ratzui et al., Wong et al., Hui et al., Chan et al., Evans et al., and Ekstedt et al. (14–20). Observational parameters are then fixed for subsequent steps. In step 2, a scale-factor (α) is fit for the corresponding forward parameters to describe the placebo effect in Cusi et al., Belfort et al., and Aithal et al. (21–23) trials. Step 3 fits the pioglitazone effect with an additional scale factor (β) on both forward (p1, p3, p5, and p7) and reverse parameters (p2, p4, p6, and p8) for just the Cusi et al. (21) trial. Step 4 fixes all previously estimated parameters to predict the outcome of the pioglitazone arm in Belfort et al. and Aithal et al. (22, 23).

The impact of pioglitazone on parameter estimates was estimated in a three-step process. First, we assumed each study exhibits a separate placebo effect and this placebo effect only impacts the forward disease progression. To capture the distribution of patients in the placebo group of each study, the parameters were fixed to the observational disease progression fitted parameters, however, the forward parameters were allowed to vary by a scale-factor (α1 α3 α5 α7). Each study has its own set of α-parameters to account for study specific differences in patient response to the trial, such as adherence to the recommended diet and exercise regimens. The second step was to similarly estimate the scale-factor change from observational fitted parameters for both the forward and reverse parameters for treatment with pioglitazone. Under the assumption that the same set of coefficients would capture all the pioglitazone data, we used the Cusi et al. data set to fit these parameters because it had the longest study end time. We then fixed the Cusi et al. parameters with their respective α-parameters and estimated a second set of scale-factors (β1–β8). Lastly, we attempted to validate the pioglitazone parameters by simulating the outcome of the Belfort et al. and Aithal et al. studies (22, 23). A flowchart describing the parameter fitting and validation sequence is shown in Figure 2.

### 2.5. Clinical trial design

Finally, we simulated the model to demonstrate how it may be useful in clinical trial design. One of the many challenges in designing a clinical trial for the treatment of NAFLD and NASH is determining the number of patients required to reach clinical significance given large interpatient and intrapatient variability in fibrosis score. The CTMC model is well-posed to address this problem since the stochastic nature of the model is agnostic to the sources of variability. The first assessment performed was to determine how the placebo response may change given different distributions of the patient population at each stage of fibrosis. Each stage of fibrosis was simulated with 100 patients for a duration of 12 months to determine the percentage of that population whose scores would improve or worsen over time.

The second assessment was to use information from model simulations to identify the minimum number of patients necessary to power a clinical trial with a drug effect similar to that of pioglitazone. To do this we simulated a minimum of 90 different initial distributions for a given number of virtual patients. The patient distribution was randomly selected according to the proportions of the initial distributions in the Cusi et al. (21) data set. For each set of data, a virtual clinical trial was simulated 200 times to generate statistics for the average change in fibrosis score, and standard deviation for that given data set. We then calculated the power for each initial distribution using a two-sample *t*-test. The number of virtual patients in each trial was varied from 5 to 125 by increments of 5.

## 3. Results

### 3.1. Observational data fitting

**Figure 3:**
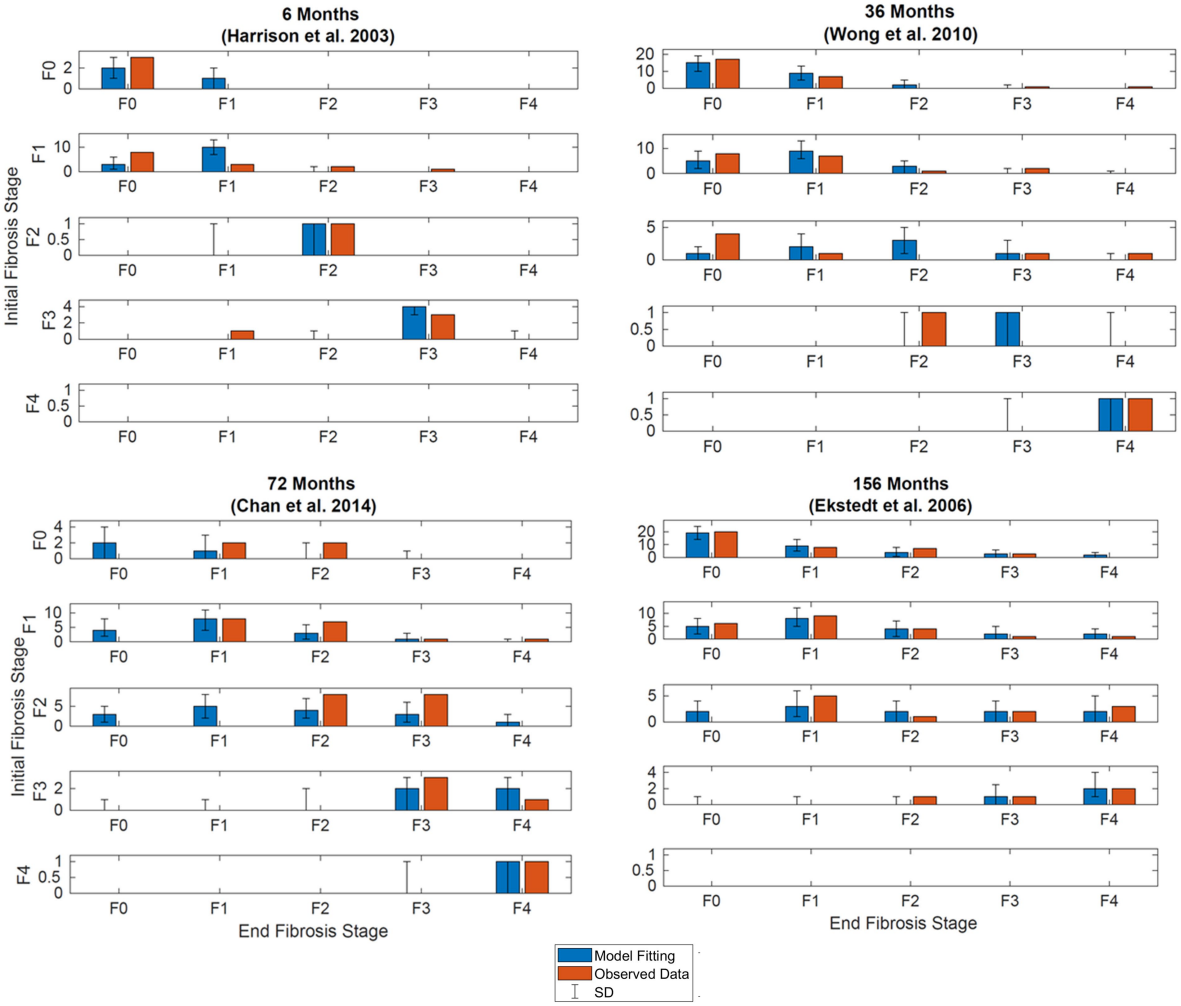
Simultaneous model fitting of observational data. Observed (orange bars) and model (blue bars). **(A)** Model fitting at 6 months. Compared to data digitized from Harrison et al. (20, p.2488). **(B)** Model fitting at 36 months. Compared to data extracted from Wong et al. (14, p.972). **(C)** Model fitting at 72 months. Compared to data extracted from Chan et al. (19, p.550). **(D)** Model fitting at 56 months. Compared to data extracted from Ekstedt et al. (17, p.871). Initial stages of fibrosis are indicated on the *y*-axis, the final stages of fibrosis for patients in the respective starting stages are shown on the *x*-axis. Error bars illustrate the standard deviation after simulating the model with the same initial distribution 500 times.

Results of the model fitting are shown in Figure 3. Observed data are presented side by side with model fittings. The model fittings performed reasonably well to capture the observed data. Upon visual inspection, 85% of the fitted data is within two standard deviations of the observed data.

### 3.2. Sensitivity analysis

**Figure 4:**
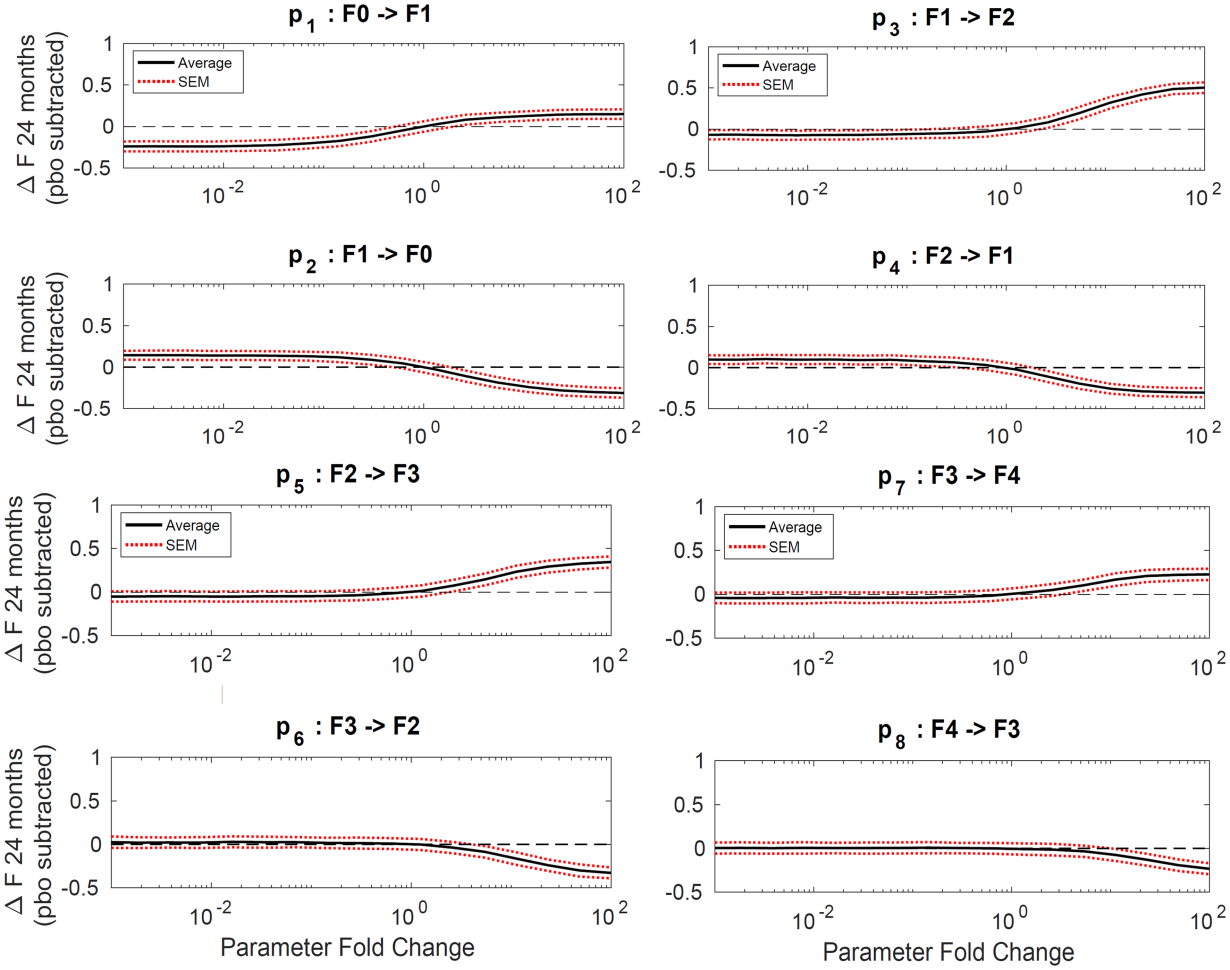
Sensitivity analysis. Plots of average change in fibrosis score vs. fold change in a single parameter while all others remain constant. Progressor fraction was fixed to the fitted value. Solid lines represent the average change in fibrosis score and the dashed lines are the standard error of the mean.

Sensitivity analysis showed the average fibrosis score was most sensitive to changes in parameters p1, p2, p3, and p4 as shown in Figure 4. The most sensitive parameter was shown to be the reverse parameter from F1 to F0, p2, a 10-fold increase results in a 0.5-point decrease in the average fibrosis score. Parameters p5, p6, p7, and p8 have less influence on the average fibrosis score since there are fewer patients represented in F3 and F4, which is typical of the published clinical trial cohorts.

### 3.3. Pioglitazone intervention

**Figure 5:**
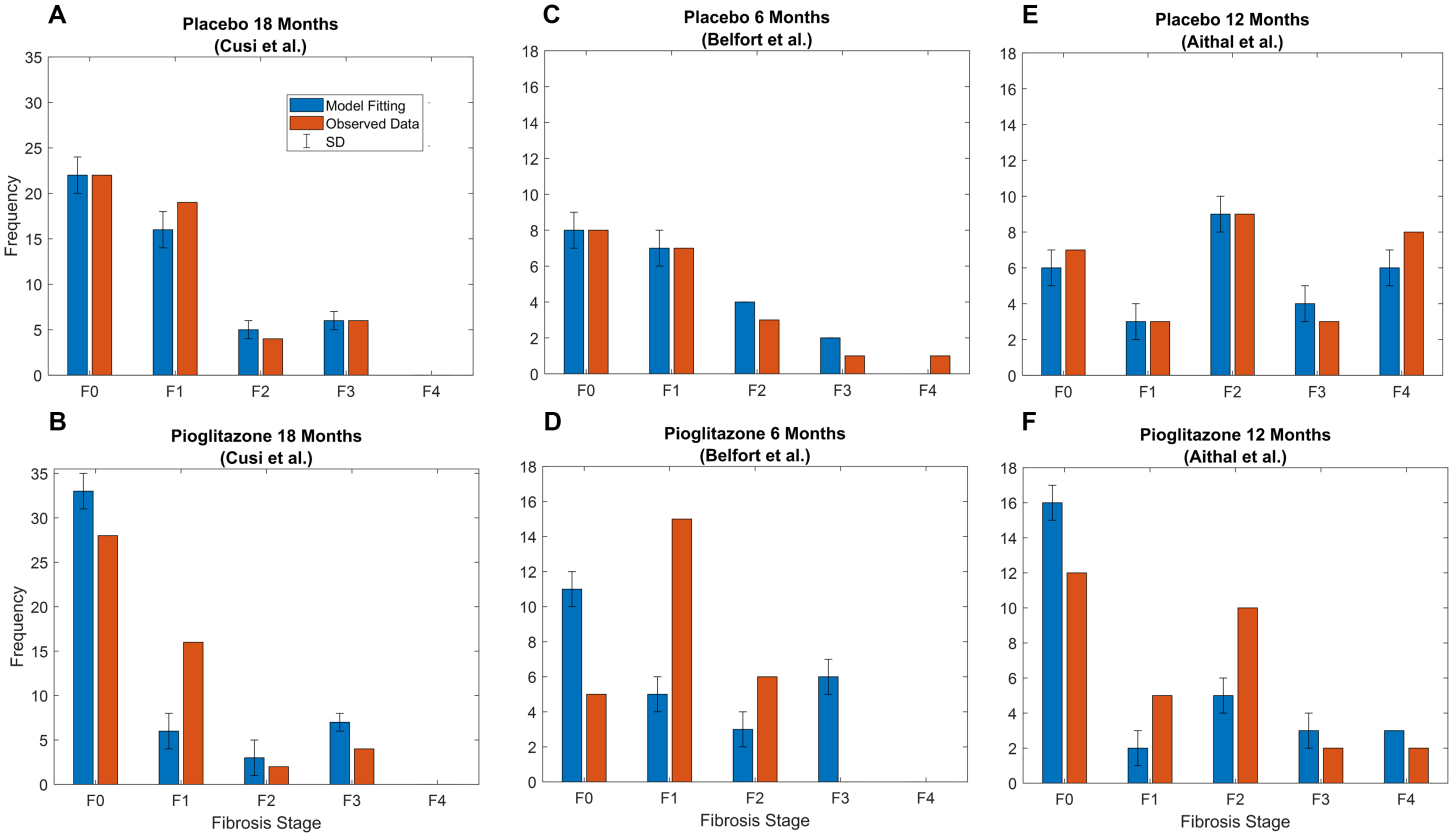
Pioglitazone simulation results. Observed (orange bars) and predicted (blue bars) data ± SD. **(A)** Fitting of Cusi et al. data after 18 months [Appendix (21)] with just α parameters, which are the fold changes for forward parameters. **(B)** Fitting for β parameters representing fold change for pioglitazone effect in the Cusi et al. study. **(C)** Fitted placebo α parameters for Belfort et al. **(D)** Fixed parameters representing the placebo effect and pioglitazone effect for Belfort et al. data extracted from p.2305 (22) after treatment. **(E)** Fitted placebo α parameters for Aithal et al. **(F)** Fixed parameters representing the placebo effect and pioglitazone effect for Aithal et al. data extracted from p.1179 (23) after treatment.

Final parameter estimates for α and β coefficients are shown in Table 3. Placebo effects (α-parameters) on fibrosis progression ranged from less than 2- to 4-fold compared to observational disease progression parameter estimates. Pioglitazone effects ranged from 2- to 30-fold for Cusi et al. data whereas some β-parameter estimates were from 25-fold to upward of 40-fold for data from Belfort et al. and Aithal et al. β-parameter estimates suggest that pioglitazone not only slows disease progression but reverses fibrosis in the liver as indicated by a faster transition to lower fibrosis scores. Figure 5 shows the model simulations compared to observed data.

**Table 3:** Final parameter estimates.

	Fitted parameters		Placebo (α)		Pioglitazone (αβ)	
Parameters	Forward	Backward	Forward	Backward	Forward	Backward
	τ (months)	τ (months)	τ (months)	τ (months)	τ (months)	τ (months)
F0 < -> F1	5.7	10	9.3	10	139	3.8
F1 < -> F2	86	51	68	51	1,750	7.6
F2 < -> F3	97	208	52	208	1,570	171
F3 < -> F4	116	526	374	526	374,000	2,140
Progressor fraction	0.64	Estimated	0.64	Fixed	0.64	Fixed

τ_n_ Reaction Time (months) 1/p_n_.

### 3.4. Clinical trial design

**Figure 6:**
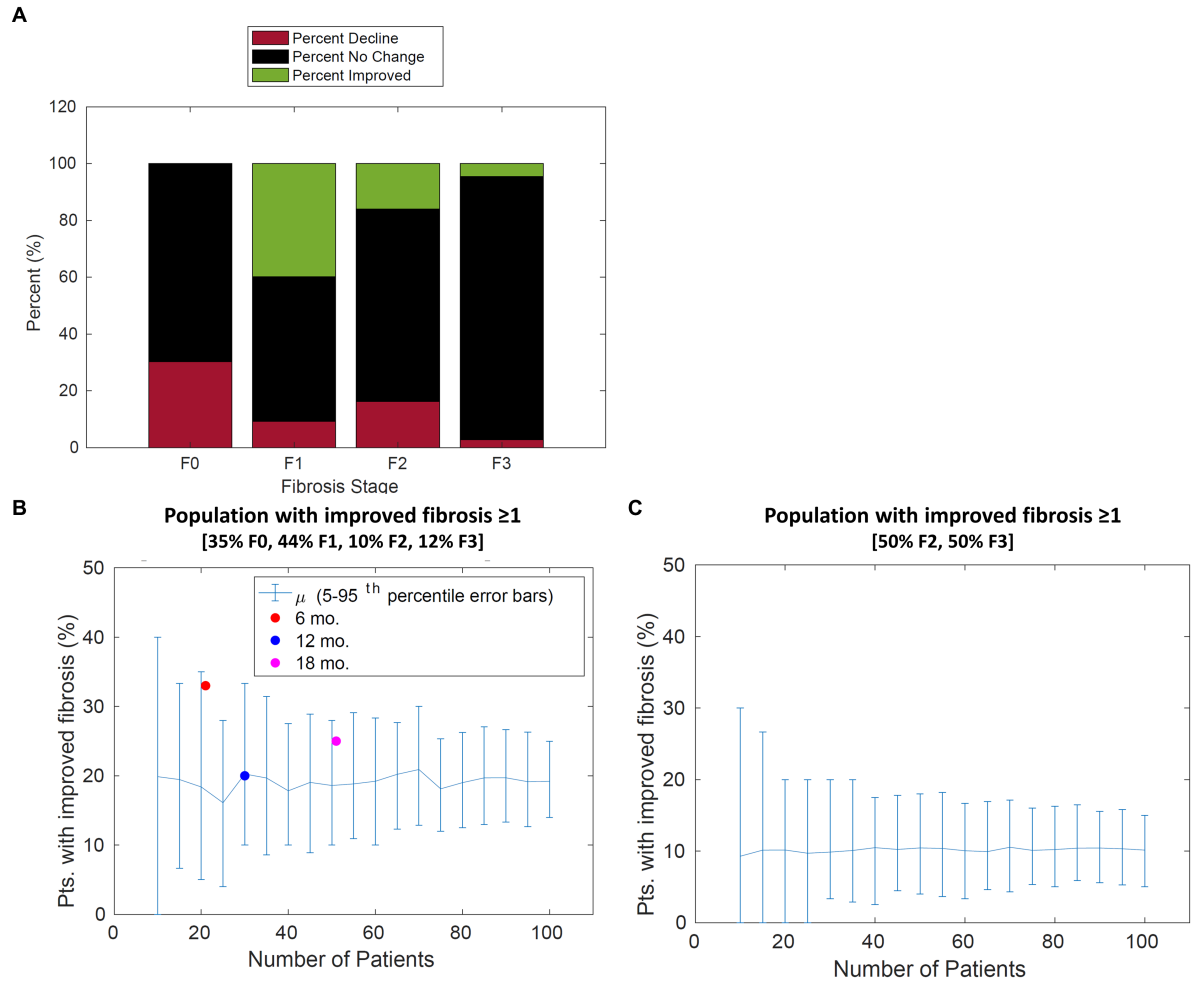
Population change in fibrosis score over 12 months. **(A)** For each stage of fibrosis, the average predicted percent of the population that improved (green bars), declined (red bars), or stayed the same (black bars). **(B)** The predicted percentage of patients with improvement in fibrosis score of 1 or more vs. the number of patients in the study for a typical patient cohort. The percentage of patients improved from Cusi et al. extracted from [Appendix, (21)], Belfort et al. extracted from p. 2305, and Aithal et al. extracted from p. 1179 (21–23) is overlaid with model simulations. **(C)** The predicted percentage of patients with improvement in fibrosis score of 1 or more vs. the number of patients in the study for a cohort with 50% F2 and 50% F3 patients. Error bars represent the 90th percentile.

The percent of patient improvement depends on the initial stage of fibrosis. The model estimates that within 1 year, 40% of patients categorized with stage 1 fibrosis will improve by one stage, whereas only 20% of patients in stage 2 will improve by 1 stage or more. These results suggest that the expected placebo effect will be dependent on the initial distribution of the patient population as shown in Figure 6.

**Figure 7:**
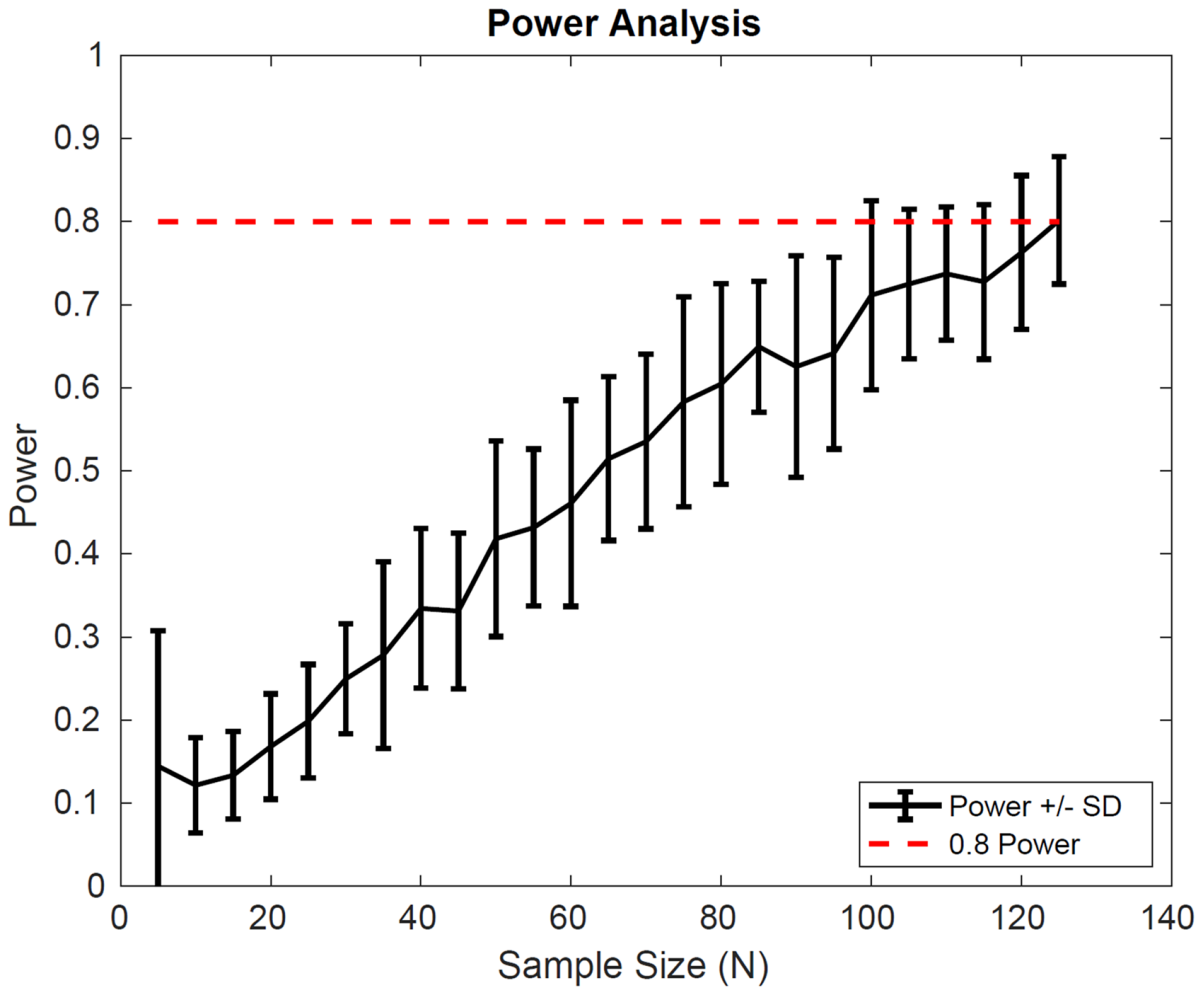
Simulated power Analysis. Power of a clinical trial vs. the number of patients in each cohort of the trial. The endpoint for this analysis was a 0.5 difference in the average fibrosis score between control and treatment groups with 80% power. Power was calculated based on two-sample *t*-test of simulated outputs.

The power analysis shown in Figure 7 suggests a sample size of 65 patients in each cohort of a clinical trial would be sufficient to detect a difference in the average fibrosis score of 0.5 between control and treatment groups with 80% power. These results assume that the drug effect is similar to pioglitazone and the patient population inclusion criteria are similar to that of Cusi et al.

## 4. Discussion

The progression of fibrosis in NAFLD and NASH is not well characterized, and surrogate plasma biomarkers have remained elusive. One approach to enhance our understanding of disease progression is to estimate the average number of stages patients’ progress in a year from paired liver biopsy studies as illustrated by Singh et al. In this systematic review, the authors estimated the fibrosis progression rate differentiating between NAFLD patients and NASH patients. They found the average progression rate from stage 0 to stage 1 was 0.07 years for patients with NAFLD and 0.14 years for patients with NASH (24). One shortcoming of this estimation method, however, is that it cannot account for the heterogeneity of disease progression observed in the population. In addition, the authors faced challenges estimating the fibrosis progression rate for stages 2–3 and 3–4 citing that the negative lower limit of the confidence intervals suggest there could be net regression of fibrosis stage. These observations motivated us to develop a modeling approach in order to quantify fibrosis progression, regression, and its associated variability.

We chose to employ a continuous-time Markov chain model as clinical data collected from histological assessment of NAFLD and NASH lends itself readily to Markov chain modeling. A CTMC model can estimate both rates of progression as well as rates of regression at each stage in fibrosis. It is necessary to include the regression mechanism as the data in literature suggests fibrosis improves in patients advised on diet and exercise, as well as patients treated with pioglitazone (25). The disadvantage of this modeling approach is that it requires sufficient data to estimate each parameter and there is limited data for patients in the later stages of fibrosis.

In our approach, we collected data from several studies reporting the fibrosis scores from paired liver biopsy studies; many of the studies overlap with the analysis performed by Singh et al. These studies consisted of patients with biopsy confirmed NAFLD or NASH and included patients with diabetes, metabolic syndrome, hypertension, reduced insulin sensitivity, and obese and non-obese patients. All patients in the studies were advised on diet and exercise but were otherwise not specifically treated for NAFLD or NASH. Using all the available information, we were able to observe reasonable fits between the model and observed data as shown in Figure 3.

The fraction of patients progressing from stage 0 was estimated to be 64%. This agrees with the raw data showing that 58% of patients that started in F0 at all time points ended in F0 as well as the Ekstedt et al. data which shows 53% of patients starting in F0 remained in F0 at 13 year follow up (17). Further work is necessary to elucidate potential biomarkers to differentiate progressors from non-progressors. The rate of progression from F0 to F1 for a progressive population was estimated to be 5.7 months or 0.47 years, which is similar to the estimation presented by Singh et al. for NASH patients (0.14 years) (24). The advantage to using a modeling approach is that we can now make predictions about a given initial distribution and leverage the stochastic property of the model to incorporate variability.

Sensitivity analysis indicates the maximum decrease in average fibrosis score by altering a single parameter is 0.5 stages. However, this does not suggest that any putative therapy designed to have a larger impact on the disease would necessarily have to exhibit poly-pharmacology. In this framework, we have made no mechanistic interpretation of the parameters and in fact the same biological process could be captured in multiple parameters (for example, collagen deposition might be encapsulated in all forward parameters). We found the parameters that have the most impact over the average fibrosis score are p_1_, p_2_, p_3_, and p_4_. Since the majority of patients in the initial distribution are in stages F0 and F1, the influence of changing p_5,_ for example, does not significantly alter the average fibrosis score. One caveat to the sensitivity analysis is also that the simulation duration was 24 months. Parameter estimates for stages F2, F3, and F4 are estimated to have average reaction times from 86 up to 116 months as shown in Table 3. This may exclude the impact of changing a parameter 100-fold like for parameters p_5_ and p_6_, which are 97 and 208 months, respectively, for a population with more patients in stages F3 and F4.

Next, we investigated the effects of placebo and pioglitazone from a clinical trial as a proof of principle for the CTMC model. Parameter estimates to capture the placebo effect suggested the recommended diet and exercise regimen in the placebo group slows disease progression. Weight loss was also observed in the placebo groups of Aithal et al. (22) and Belfort et al. (23) studies. Pioglitazone, however, has notable effects on both slowing disease progression and improving the fibrosis score, as suggested by alterations in both the forward and reverse parameters; i.e., the data could not be captured without accounting for the reversal mechanism corresponding to a decrease in fibrosis score. These results also agree with the sensitivity analysis; we found p_1_ reaction time increased by 10-fold, and p_4_ reaction time decreased by 6-fold resulting in a decrease of 0.5 for the average fibrosis score with pioglitazone. Future work is necessary to validate the parameter estimates for the impact of pioglitazone. Cusi et al. reported only initial and final distributions for the placebo and treated groups—if the data had been reported categorically for each stage, such as Ekstedt et al. (17), then our estimates might be more robust. Perhaps due to this limitation, estimating individual placebo effects and simulating the results for data presented in Belfort et al. and Aithal et al. did not capture the data, as indicated in Figures 5D,F. Perhaps more crucially, the patient populations recruited for each trial were different; for example, the Aithal et al. trial excluded patients with type 2 diabetes. Additionally, the sample size for each study was relatively low, with only 20 or 30 patients. This approach applied to richer larger datasets, with individual level data, may be able to elucidate treatment effects with more clarity.

Model simulations were performed to gain insight on disease progression variability to inform clinical trial design. Based on our findings from parameter fitting, sensitivity analysis revealed the expected placebo response (i.e., the percentage of patients improving) may depend on the initial distribution. Simulations predict the placebo response will be greater if a larger number of patients in stage F1 are included due to the shorter transition time. The placebo response ranges from 20% of patients improve by one stage or more for a typical distribution (35% F0, 44% F1, 10% F2, 12% F3, and 0% F4) compared to 13% for a distribution containing 50% F2 and 50% F3 patients (Figures 6B,C).

Finally, we simulated the outcome of a clinical trial multiple times for a range of patient sample sizes to calculate the average power of a study, with a random initial distribution. We found a sample size of 65 patients in each group would be sufficient to power a study with 80% power to detect a difference of 0.5 in average fibrosis score with a significance of 0.05. The clinical trial design, however, will depend on the drug effect and a clearly defined patient population.

## 5. Conclusion

The CTMC modeling approach enabled us to estimate forward and reverse parameters for fibrosis in NAFLD and NASH. We found that intervening at earlier stages of fibrosis is more likely to improve the average fibrosis score. This finding is confirmed in clinical trials of pioglitazone (21–23). In addition, model fitting suggests pioglitazone plays a role in reversing fibrosis progression, though, these results may be caveated by small sampling size, biopsy sampling variability, and pathologist variability. Based on our analysis, a study powered at 80% to detect a − 0.5 change in average fibrosis score, as observed by Cusi et al., would require a larger sample size to reduce the risk of a type 1 error. The modeling work presented here is well-suited for better understanding the placebo response for a clinical trial. We found the CTMC model reproduces the trends in the data and broadly recapitulates the variability associated with fibrosis score.

Future applications of this model could include bridging a quantitative systems pharmacology (QSP) model to the CTMC model. A QSP model would facilitate connecting mechanistic drivers of disease progression to clinically measured fibrosis score. To combine the CTMC model with a more mechanistic model, it may be necessary to incorporate variability in the reaction rates. This can be accomplished by back-calculating the integrals of the reaction rates (13) in order to couple disease progression to a more mechanistic model of underlying disease pathogenesis. As more data from ongoing clinical studies is published, the model will become more powerful at predicting fibrosis progression for NAFLD/NASH patients.

## Data availability statement

The data used in this study are available in PubMed Central with the following accession numbers: PMID 14638353, PMID 10833486, PMID 20581244, PMID 15709991, PMID 25060399, PMID 12195000, PMID 17006923, PMID 27322798, PMID 17135584, PMID 18718471.

## Author contributions

RA contributed to conception and design of the study. LM and RA executed the analysis. All authors contributed to the article and approved the submitted version.

## Funding

The authors declare that this study received funding from Pfizer, Inc. The funder had the following involvement with the study: Pfizer, Inc. is the employer of LM, RA, and CJM. Pfizer had no influence over the study design, analysis, interpretation or decision to submit for publication.

## Conflict of interest

Pfizer Inc. supported the research by LM, RA, and CJM. LM, RA, and CJM are employees of Pfizer.

## Publisher’s note

All claims expressed in this article are solely those of the authors and do not necessarily represent those of their affiliated organizations, or those of the publisher, the editors and the reviewers. Any product that may be evaluated in this article, or claim that may be made by its manufacturer, is not guaranteed or endorsed by the publisher.
